# A Fatal Case of Hypertriglyceridemia-Induced Acute Pancreatitis in a Patient With Diabetic Ketoacidosis

**DOI:** 10.7759/cureus.14968

**Published:** 2021-05-11

**Authors:** Bhavya Narala, Amna Al-Tkrit, Sharoon David, Harith Alataby, Jay Nfonoyim

**Affiliations:** 1 Internal Medicine, Richmond University Medical Center, Staten Island, USA; 2 Internal Medicine, Jamaica Hospital Medical Center, Richmond Hill, USA; 3 Pulmonary and Critical Care, Richmond University Medical Center, Staten Island, USA

**Keywords:** diabetic ketoacidosis, acute pancreatitis, hypertriglyceridemia, acute respiratory distress syndrome, plasmapheresis, diabetes, insulin, serum lipase

## Abstract

Diabetic ketoacidosis (DKA) with coexisting hypertriglyceridemia-induced acute pancreatitis is a rare yet potentially life-threatening condition. This report describes a patient with no history of diabetes who presented with DKA and coexisting acute pancreatitis secondary to severe hypertriglyceridemia. The patient did not respond to standard DKA management or plasmapheresis, developed acute respiratory distress syndrome (ARDS), and eventually expired.

## Introduction

Diabetic ketoacidosis (DKA) is a serious complication of diabetes and may rarely be the initial manifestation of type 2 diabetes. Moreover, in rare cases, DKA may be associated with severe hypertriglyceridemia, which can result in the development of acute pancreatitis. The overlapping clinical features make it difficult to establish an early diagnosis of this complication, which can delay proper management and result in significant morbidity and mortality. This potentially life-threatening triad of DKA, hypertriglyceridemia, and acute pancreatitis is rare and has not been widely described in the medical literature. In this report, we describe a case of hypertriglyceridemia-induced acute pancreatitis in a patient with DKA. The disorder did not improve with treatment and resulted in the death of the patient.

## Case presentation

A 37-year-old man presented to the emergency department of our hospital with complaints of nausea, vomiting, and left-sided abdominal pain. One day ago, the patient developed nausea and had 4-5 episodes of non-bloody, non-bilious vomiting but was still tolerating oral intake at that time. However, on the day of hospital admission, her wife found him to be extremely lethargic and tachypneic, which prompted her to bring him to the emergency department. The patient reported that he had been experiencing abdominal pain, excessive thirst, and frequent urination for the past few days but denied any history of fever, chills, headache, dizziness, chest pain, shortness of breath, diarrhea, or constipation. The patient had a past medical history of diverticulitis but had no history of diabetes. He did not have a primary care physician and was not taking any prescription medications. He only took over-the-counter ibuprofen, when needed. The patient drank 2 to 3 six-pack of beer and smoked two packs of cigarettes every week but denied any illicit drug use. 

**Figure 1:**
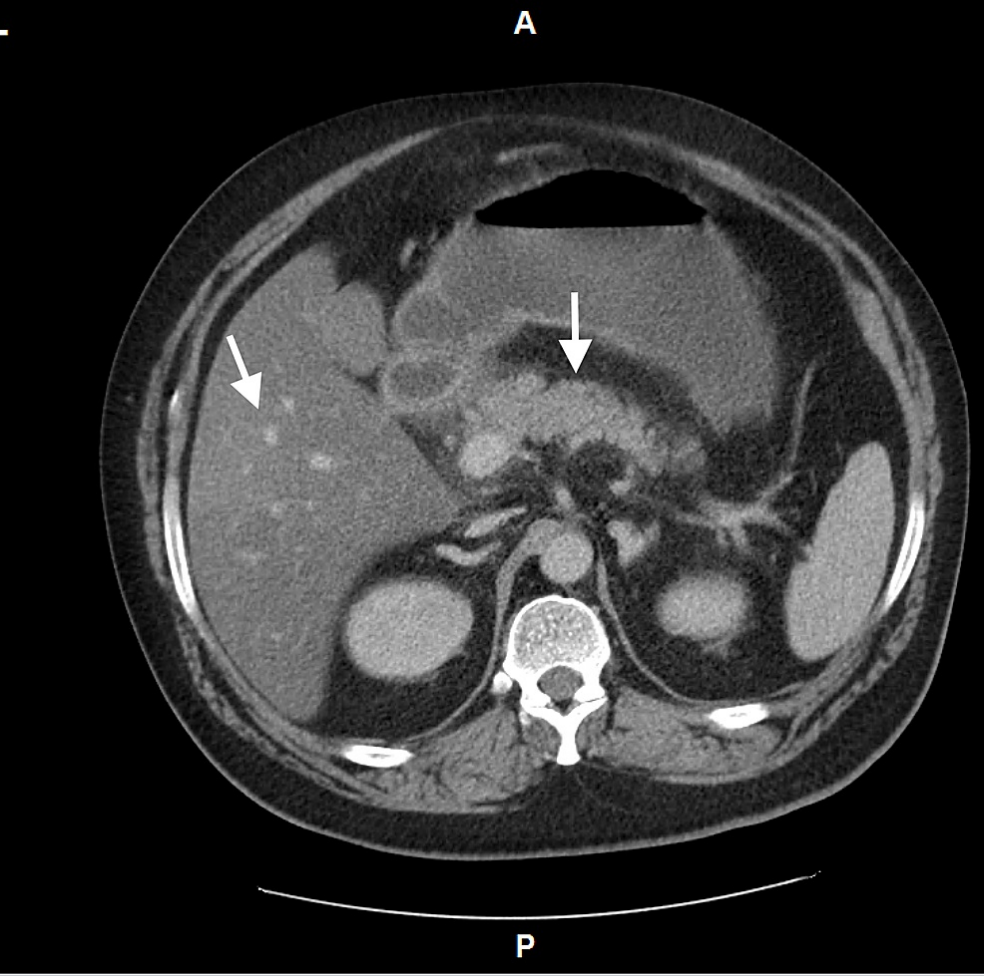
CT abdomen/pelvis with contrast exhibiting mild pancreatitis with hepatic steatosis.

In the emergency department, his vital signs included a blood pressure of 105/61 mmHg, heart rate of 132 beats/min, respiratory rate of 30 breaths/min, and oxygen saturation of 98% on a non-rebreather mask. On examination, the patient was awake, alert, and oriented, but was lethargic and appeared to be in moderate respiratory distress. He was found to have severe DKA with a blood sugar level of 692 mg/dL and an anion gap of 28. Urinalysis was positive for ketones. Intravenous fluids, morphine, metronidazole, levofloxacin, and regular insulin were administered. His serum lipase was found to be 3.013 U/L. A CT scan of the abdomen and pelvis was performed and showed mild pancreatitis with reactive chemical duodenitis, diverticulosis coli, and hepatomegaly with diffuse hepatic steatosis (Figure 1). In addition, the patient had a bicarbonate level of 4 mEq/L and was severely acidotic with a pH of 6.98. Insulin and bicarbonate infusions were started.

**Figure 2:**
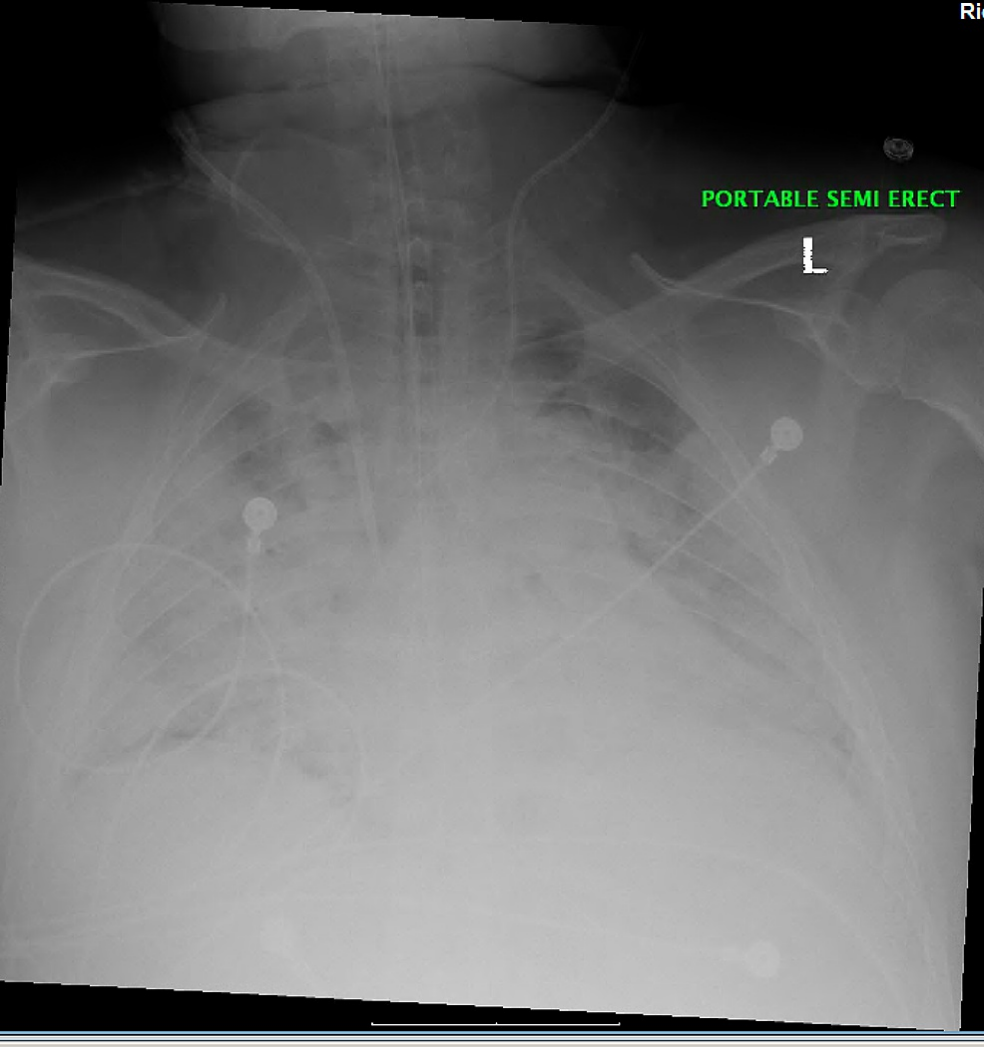
CXR exhibiting ARDS. CXR: chest X-ray; ARDS: acute respiratory distress syndrome.

Later, the patient had to be intubated secondary to increasing lethargy and respiratory distress. His blood sugar levels showed some improvement but still remained around 400 mg/dL. The lipid panel showed a markedly elevated triglyceride level of more than 2,000 mg/dL. On the next day, plasmapheresis was performed and the triglyceride levels were reduced to 400 mg/dL. His blood pressure remained low, and so, norepinephrine was started. However, the blood pressure was still not stabilized, and hence, phenylephrine followed by vasopressin had to be started. The next day, he developed acute respiratory distress syndrome (ARDS) (Figure 2) and ARDS protocol was initiated. The patient was receiving multiple medications for sedation, including propofol, midazolam, vecuronium, and fentanyl; and also received a course of metronidazole and aztreonam, although the source of infection was not identified. In addition, electrolyte repletion with calcium, phosphorous, magnesium, and potassium supplementation was performed.

**Figure 3:**
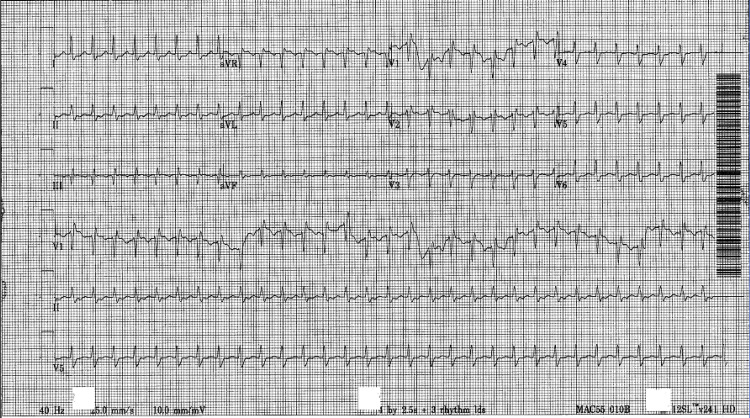
Supraventricular tachycardia.

A day prior to expiration, the patient was noted to have a heart rate of 130 beats/min. He was on a maximum dose of three vasopressors along with a bicarbonate infusion, an insulin infusion, and multiple medications for sedation. He was also noted to have a fever of 105 degree F that did not resolve with the use of ice packs and recurring doses of acetaminophen. During that night, his heart rate reached up to 160-180 beats/min. EKG revealed supraventricular tachycardia (SVT) (Figure 3). Adenosine and cardioversion were attempted with no resolution of symptoms. On the following day, the patient was found to be in asystole. Cardiopulmonary resuscitation (CPR) was performed but failed to revive the patient.

## Discussion

Diabetic ketoacidosis (DKA) is a potentially life-threatening condition that is characterized by the biochemical triad of hyperglycemia, ketonemia, and metabolic acidosis with an elevated anion gap. It is a serious complication of decompensated diabetes and is typically seen in patients with type 1 diabetes, as a consequence of an absolute deficiency of insulin and a concomitant increase in counter-regulatory hormones. However, DKA may also occur in patients with type 2 diabetes who have a relative deficiency of insulin [1,2]. According to a recent systematic review, the incidence of DKA in patients with type 1 diabetes ranges from 0 to 56 cases per 1,000 person-years in different geographic areas [3]; whereas, cohort studies of adults with type 2 diabetes have reported the incidence of DKA to be < 2 cases per 1,000 person-years in these patients [4]. Less often, DKA may be the presenting manifestation of type 2 diabetes. This heterogeneous condition is known as ketosis-prone diabetes and is characterized by the development of DKA or unprovoked ketosis in patients who lack the typical phenotype of autoimmune type 1 diabetes [5].

Any illness or physiological stress may act as a precipitating factor for the development of DKA; however, the presence of infection, particularly a urinary tract infection (UTI) or gastroenteritis, and inadequate insulin therapy are the two most common triggers [1,2]. Patients with DKA typically present with nausea, vomiting, and abdominal pain. Polydipsia, polyuria, and dehydration may also be present. Hypotension and altered sensorium can be seen in severe cases. Examination may reveal the presence of rapid shallow Kussmaul breathing and fruity odor of the breath. [6] Three main factors used to diagnose DKA include plasma glucose levels greater than 250 mg/dL, the presence of ketones in urine or serum, and metabolic acidosis with a serum bicarbonate level less than 18 mEq/L and/or pH below 7.30 [3].

An absolute or relative deficiency of insulin coupled with an increase in the concentration of counter-regulatory hormones, including glucagon, cortisol, catecholamines, and growth hormone, is the primary mechanism involved in the pathophysiology of DKA. It causes an increase in hepatic gluconeogenesis and glycogenolysis, resulting in increased hepatic glucose production, as well as a reduction in the utilization of glucose in peripheral tissues. Insulin deficiency also causes activation of hormone-sensitive lipase that increases lipolysis in adipose tissue and leads to a subsequent increase in the release of free fatty acids (FFAs). These FFAs are oxidized by the liver resulting in the production of ketone bodies. The increased production of ketone bodies leads to a decrease in serum bicarbonate and metabolic acidosis [7]. FFAs are raw materials for the production of very low-density lipoproteins (VLDL), and thus, an increased release of FFAs accelerates the formation of VLDL by the liver. Furthermore, insulin deficiency is also associated with a reduction in the activity of peripheral tissue lipoprotein lipase (LPL), an enzyme responsible for triglyceride metabolism. Hence, the removal of VLDL and chylomicrons from the plasma is decreased, resulting in the development of hypertriglyceridemia [8,9]. 

Hypertriglyceridemia is an uncommon but well-recognized cause of acute pancreatitis and has been found to be the underlying etiology in 2%-4% of the patients. A triglyceride level of more than 1,000 mg/dL is typically associated with the development of acute pancreatitis; however, the exact level above which acute pancreatitis may occur is unknown and may vary among the patients. [10] The exact mechanism by which hypertriglyceridemia causes acute pancreatitis is not yet fully understood; however, two theories have been put forward to explain the pathophysiology. According to one of these theories, the metabolism of excess triglycerides, transported as triglyceride-rich lipoprotein particles, known as chylomicrons, occurs in the vascular bed of the pancreas. This results in the release of high levels of free fatty acids (FFAs), which exceed the binding capacity of plasma albumin. The unbound, cytotoxic free fatty acids self-aggregate into micellar structures and can cause damage to the acinar cells, vascular endothelium of pancreatic capillaries, and platelets. The resultant ischemia subsequently produces an acidic environment, which further increases free fatty acid toxicity by causing activation of trypsinogen, and this, in turn, triggers acute pancreatitis [11,12]. The second hypothesis suggests that the elevated levels of chylomicrons cause plasma hyperviscosity. The increase in plasma viscosity leads to the plugging of pancreatic capillaries and ischemia. This enhances acidosis and eventually triggers acute pancreatitis. It may be likely that both these proposed mechanisms play a contributory role in the development of hypertriglyceridemia-induced acute pancreatitis [11]. 

Moderate elevations of triglyceride levels are common in DKA; however, severe hypertriglyceridemia with triglyceride levels greater than 1,000 mg/dL is a rare complication that may increase the risk of developing acute pancreatitis [9]. One study found an association between DKA and acute pancreatitis in 11% of the cases [13]. However, the classical triad of DKA with hypertriglyceridemia-induced acute pancreatitis has been reported in only 4% of the patients. Moreover, this is an extremely rare initial presentation of diabetes in adults [14]. The clinical presentation of hypertriglyceridemia-induced acute pancreatitis is similar to that of acute pancreatitis from other causes and includes abdominal pain, elevated levels of pancreatic enzymes, and typical radiologic findings of acute pancreatitis [10]. However, the identification of acute pancreatitis in patients with DKA may be challenging for a number of reasons. Firstly, epigastric or abdominal pain is a common finding in DKA, and thus, DKA may mask coexisting acute pancreatitis. Secondly, one-fourth of the patients with DKA may have elevated levels of serum amylase and lipase in the absence of any clinical or radiological signs of acute pancreatitis. Furthermore, the pancreatic enzyme levels may remain normal in some patients with DKA and concurrent acute pancreatitis [15]. Patients with DKA and severe hypertriglyceridemia may also have markedly decreased serum sodium and chloride levels, which may result in a delay in proper management. This may be due to laboratory interference as hyperglycemia or hyperlipidemia can result in significantly lower sodium and chloride levels, if an indirect, rather than a direct, ion-selective electrode method is used to measure these ions [16].

In light of these challenges, it is important to maintain a high index of suspicion for the early identification of acute pancreatitis in any patient with DKA. For instance, in patients with continuous abdominal pain and nonspecific elevations of serum amylase and/or lipase, further laboratory evaluation or CT imaging of the abdomen may be considered [17]. Measurement of serum lipase is superior to serum amylase analysis, and an elevation of serum lipase more than three times of upper normal limit is considered to be 100% sensitive and 99% specific for acute pancreatitis [16]. The presence of lactescent serum also strongly suggests a diagnosis of hypertriglyceridemia-induced acute pancreatitis [11]. Finally, the presence of unusually low levels of serum sodium and chloride, in the presence of lipemic blood, should point towards a diagnosis of hypertriglyceridemia-induced acute pancreatitis in a patient with worsening DKA [16]. If the patient is in a hyponatremic state, the possibility of pseudohyponatremia should be considered and overtreatment with hypertonic saline should be avoided [17].

The triad of DKA, hypertriglyceridemia, and acute pancreatitis has been found to have a worse prognosis in comparison to simple acute pancreatitis and can lead to devastating consequences. A higher rate of multiorgan failure, parenteral nutrition requirement, and inpatient mortality, as well as a longer hospital length of stay, have been reported in patients with this triad [18]. Patients with DKA who develop acute pancreatitis also have a higher risk of developing acute kidney injury (AKI), ileus, shock, acute respiratory distress syndrome (ARDS), and systemic inflammatory response syndrome (SIRS), as compared to patients without DKA [19]. Hence, early diagnosis and prompt initiation of treatment are critical. The management of patients with DKA and hypertriglyceridemia-induced acute pancreatitis usually involves standard DKA management, as insulin deficiency is the primary mechanism of hypertriglyceridemia. The treatment includes aggressive intravenous fluid resuscitation with 0.9% normal saline initially, followed by the use of 5% dextrose with 0.45% normal saline, the administration of insulin as a continuous infusion before transitioning to subcutaneous route, and electrolyte repletion. Bowel rest is required, and once the patient is tolerating a clear liquid diet, a fibrate should be initiated to lower the triglyceride levels. However, in patients with severe hypertriglyceridemia, especially those with organ failure, standard care may be insufficient and urgent plasmapheresis may be required [9]. The removal of chylomicrons, pro-inflammatory cytokines, and proteases, and a decrease in the plasma hyperviscosity are thought to be the different mechanisms by which plasmapheresis helps lower triglyceride levels in these patients [20]. Heparin and omega oils have also been reported as successful treatment options for the management of patients with DKA and severe hypertriglyceridemia [9].

Severe hypertriglyceridemia resulting in the development of acute pancreatitis is a rare yet potentially fatal complication of diabetic ketoacidosis (DKA). Our patient who presented with a history of abdominal pain, nausea, and vomiting, was found to have severe DKA. An elevated level of serum lipase and radiologic evidence helped establish the diagnosis of coexisting acute pancreatitis, and a triglyceride level > 2,000 mg/dL confirmed the classical triad of DKA, hypertriglyceridemia, and acute pancreatitis. Standard treatment for DKA was administered but failed to cause a significant improvement in the patient’s clinical status. Plasmapheresis led to a reduction in the patient’s triglyceride levels; however, his condition continued to deteriorate and he developed ARDS, and eventually expired. This case highlights the significance of being mindful of the possibility of hypertriglyceridemia-induced acute pancreatitis in patients with DKA, as early recognition of this complication has important implications for the management and prognosis of the patient. Further research is warranted to gain more knowledge regarding the exact pathogenesis of this triad and to establish definitive treatment guidelines.

## Conclusions

DKA is rarely associated with the development of severe hypertriglyceridemia, which can subsequently lead to the development of acute pancreatitis. This is a potentially life-threatening condition, and thus, requires swift diagnosis and treatment. However, establishing an early diagnosis may be challenging due to the overlapping signs and symptoms. Therefore, it is important for physicians to be mindful of this rare triad in patients with severe DKA. Insulin administration and conservative management may result in clinical improvement; however, plasmapheresis may be required in severe cases. DKA patients with hypertriglyceridemia-induced acute pancreatitis have a poorer prognosis as compared to patients with simple acute pancreatitis.
